# The relationship between fat mass and obesity associated gene polymorphism rs9939609 and resting cerebral blood flow in a midlife sample with overweight and obesity

**DOI:** 10.3389/fnhum.2022.904545

**Published:** 2022-08-22

**Authors:** Chelsea M. Stillman, John M. Jakicic, Renee J. Rogers, Kathryn A. Roecklein, Grant Barrett, Chaeryon Kang, Kirk I. Erickson

**Affiliations:** ^1^Department of Psychology, University of Pittsburgh, Pittsburgh, PA, United States; ^2^Division of Physical Activity and Weight Management, Department of Internal Medicine, University of Kansas Medical Center, Kansas City, MO, United States; ^3^Wondr Health, Dallas, TX, United States; ^4^Department of Biostatistics, University of Pittsburgh, Pittsburgh, PA, United States; ^5^PROFITH “PROmoting FITness and Health Through Physical Activity” Research Group, Sport and Health University Research Institute (iMUDS), Department of Physical and Sports Education, Faculty of Sport Sciences, University of Granada, Granada, Spain; ^6^AdventHealth Research Institute, Neuroscience Institute, Orlando, FL, United States

**Keywords:** clinical trial, brain, cerebral blood flow, obesity, FTO (fat mass and obesity associated) gene

## Abstract

**Background:**

The single nucleotide polymorphism (SNP) rs9939609 in the fat mass and obesity associated fat mass and obesity associated gene (FTO) gene has been linked with increased BMI in adults. Higher BMI has been associated with poor brain health and may exert deleterious effects on neurocognitive health through cerebral hypoperfusion. However, it is unclear if there is a relationship between the FTO genotype and cerebral perfusion, or whether FTO genotype moderates the effects of weight loss on cerebral perfusion. Using data from a randomized controlled behavioral weight loss trial in adults with overweight and obesity, we tested (1) whether carriers of the A allele for FTO rs9939609 demonstrate different patterns of resting cerebral blood flow (rCBF) compared to T carriers, and (2) whether the FTO genotype moderates the effects of weight loss on rCBF. We hypothesized that carriers of the A allele would exhibit lower resting CBF in frontal brain areas compared to T/T homozygotes at baseline, and that intervention-induced weight loss may partially remediate these differences.

**Methods and results:**

One hundred and five adults (75.2% female, mean age 44.9 years) with overweight or obesity were included in the analyses. These participants represent a subsample of participants in a larger randomized controlled trial (NCT01500356). A resting pseudo-continuous arterial spin labeling (pCASL) scan was acquired to examine rCBF. Age, sex, and BMI were included as covariates. At baseline, A carriers had greater rCBF in a diffuse cluster extending into the brainstem, motor cortex, and occipital lobe, but lower perfusion in the temporal lobe. We found no evidence that FTO moderated the effect of the intervention group assignment on rCBF changes.

**Conclusion:**

Overall, these results indicate that (a) individual variation in rCBF within a sample with overweight and obesity may be attributed to a common FTO variant, but (b) a weight loss intervention is effective at increasing rCBF, regardless of FTO genotype.

## Introduction

The prevalence of overweight and obesity is increasing worldwide due to changes in lifestyles that influence physical activity and eating behavior. Overweight is defined by a body mass index (BMI) of 25 to < 30 kg/m^2^ and obesity is defined by a BMI of ≥30 kg/m^2^ (“Clinical Guidelines on the Identification, Evaluation, and Treatment of Overweight and Obesity in Adults–The Evidence Report.” National Institutes of Health, 1998). The public health effects of obesity are widespread, including but not limited to an increased risk of stroke, coronary heart disease, hypertension, type 2 diabetes, cancer, and musculoskeletal disorders (Pi-Sunyer, 2009). Obesity is also a recognized risk factor for cognitive decline in late life (Gustafson et al., 2003; Raji et al., 2009; Ho et al., 2010a).

Overweight or obesity in adulthood may pose a particular threat to brain health. Higher BMI in mid-late adulthood is associated with ischemic changes in cerebral white matter, reduced cerebral blood flow, temporal lobe atrophy, and a higher risk of Alzheimer’s disease (Gustafson et al., 2004; Taki et al., 2008; Raji et al., 2009; Willeumier et al., 2011). These indicators of poor brain health may be precipitated by overweight- and obesity-associated declines in cardiovascular health and subsequent disruptions in cerebral blood flow (Espeland et al., 2018). In fact, cerebral perfusion in certain patient populations is moderated by inter-individual variability in weight status. For example, cerebral hypoperfusion is common in patients with heart failure, but it is exacerbated in heart failure patients with overweight or obesity compared to their healthy-weight counterparts and accelerates cognitive decline in this high risk subgroup (Alosco et al., 2012). Despite the consistent negative links between BMI, vascular health, and rCBF, there is still a poor understanding of factors that contribute to variance in rCBF within adults with overweight and obesity.

Common genetic factors may play an important role in regulating both body weight and rCBF. Over 50% of the variance in BMI is heritable, and a common variation in the fat mass and obesity associated gene (FTO) accounts for most inter-person variance in body weight (Loos and Bouchard, 2008; Wardle et al., 2008; Walley et al., 2009). Specifically, the single nucleotide polymorphism (SNP) rs9939609 in the FTO gene–a thymine (T) to adenine (A) substitution–increases obesity risk (Qi et al., 2014).

There is reason to suspect that FTO may also be a predictor of brain health outcomes. The FTO A allele is associated with reduced global brain volume with regional specificity to the frontal and occipital lobes (Ho et al., 2010b; Vainik et al., 2018). FTO genotype is also associated with individual variability in physiological responses (e.g., weight loss) to diet/lifestyle interventions in populations with overweight and obesity (Xiang et al., 2016). Specifically, carriers of the obesity-predisposing A allele experience greater weight loss in response to behavioral interventions compared to T/T homozygotes (Xiang et al., 2016). This pattern of evidence suggests that common single genetic variants, such as FTO genotype, may moderate both weight status as well as the effectiveness of behavioral interventions targeting body weight. The reported relationships between FTO and body weight, and between FTO and brain volume makes FTO a candidate for explaining variance in other aspects of brain health, such as rCBF.

The present study conducted secondary data analysis from a behavioral weight loss clinical trial to examine the relationship between the FTO rs9939609 and rCBF in adults with overweight or obesity. We hypothesized that individuals carrying the obesity predisposing A allele would exhibit lower resting CBF in the frontal lobe, a region shown to be sensitive to both obesity and genetic variation in FTO (Ho et al., 2010b; Alosco et al., 2012; Olivo et al., 2016; Vainik et al., 2018), compared to T homozygotes. A secondary aim of the present study was to examine if FTO moderates the effects of a 12-month weight loss intervention on rCBF. We have previously reported an increase in rCBF following the weight loss intervention in this sample, independent of intervention group (Stillman et al., 2021). Here, we expand upon this earlier work by examining whether FTO genotype is associated with baseline differences in rCBF, and whether intervention-related changes in rCBF are moderated by FTO genotype.

## Materials and methods

### Participants

Participants were recruited from a randomized clinical trial examining the influence of physical activity and weight loss on cardiac health (NCT01500356; R01HL103646; PI: Jakicic). An ancillary project, called the Weight Loss Intervention for Neurocognition (WIN) Trial, examining whether the parent intervention modified brain outcomes (R01 DK095172; PI: Erickson) was conducted in 125 participants (98 female, 27 male) recruited from the parent study. Participants were between the ages of 22–55 (Mean ± *SD* = 44.4 ± 8.58 years) and had a BMI ranging from 25 to < 40 kg/m^2^ (M ± *SD* = 32.22 ± 3.96). As previously reported (Rogers et al., 2019; Jakicic et al., 2022), participants were excluded from the parent trial for the following reasons: (1) self-reporting ≥ 60 min per week of structured moderate-to-vigorous intensity physical activity, (2) weight loss of ≥5% within the prior 6 months, or a history of bariatric surgery, (3) history of cardiometabolic disease, diabetes mellitus, or cancer, (4) taking medication that could affect heart rate or blood pressure, (5) taking medication that could influence body weight, (6) treatment for psychological conditions that included medication or counseling, (7) currently pregnant, pregnant within the prior 6 months, or planning a pregnancy within the next 12 months, (8) planning on geographical relocation outside of the region within 12 months, (9) inability to comply with the components of the interventions, (10) or had a contraindication that would prohibit magnetic resonance imaging (MRI) scanning.

This study was approved by the University of Pittsburgh Institutional Review Board in accordance with the Declaration of Helsinki. Eligible participants provided written informed consent and completed an MRI including a pCASL sequence within 4 weeks of starting the 12-month intervention, and then again following the intervention. Participants were randomized into one of three intervention groups: Diet-only, Diet + Moderate Exercise, and Diet + High Exercise. Details of the intervention groups’ activities are described in previous publications (Peven et al., 2020; Stillman et al., 2021; Jakicic et al., 2022). Briefly, participants randomized to the Diet-only (DIET) group (*N* = 50) were prescribed an energy restricted diet of 1,200–1,800 kcal/d based on baseline body weight with instructions not to alter their physical activity. The Diet + Moderate Exercise (Diet + MODEX) group (*N* = 30) was prescribed the same diet as the Diet-only group and was also prescribed to add 5 days/wk of physical activity that started at 100 min/week and progressed to 150 min/week by the 9th week of the intervention. The Diet + High Exercise (*N* = 45) (Diet + HIGHEX) group was prescribed the same diet as the Diet-only group and was also prescribed to add 5 days/wk of physical activity that started at 100 min/wk and progressed to 250 min/week by the 25th week of the weight loss intervention.

### Weight assessment

Weight was assessed in duplicate on a calibrated digital scale (Tanita Digital Scale, Model #WB-110A) to the nearest 0.1 kg and height was measured in duplicate on a calibrated wall-mounted stadiometer (Perspective Enterprises, Inc.) as previously described (Rogers et al., 2019; Jakicic et al., 2022). Measurements were taken at baseline, at 6-months (intervention midpoint), and 12 months. Measures of weight and height were used to compute BMI (kg/m^2^).

### Genotyping

DNA was extracted from saliva samples using the QIAamp DNA extraction kit (Qiagen) following the manufacturer’s suggested protocol. Participants were genotyped with real-time PCR amplification, followed by high-resolution melt analysis (HRMA; Vossen et al., 2009). The fragment containing rs9939609 was designed using Primer3 (Kõressaar et al., 2018), and PCR product was assessed by gel electrophoresis. The PCR was performed using precision melt supermix for HRM analysis including dNTPs, iTaq™ DNA polymerase, MgCl_2_, EvaGreen dye, and stabilizers (BioRad; Hercules, California, United States). The thermal cycling reactions consisted of initial denaturation for 1 min at 94°C, followed by 40 cycles of denaturation at 94°C for 10 s, annealing at 61°C for 20 s and extension at 72°C for 20 s, followed by HRMA.

### Magnetic resonance imaging acquisition

To quantify rCBF, pseudo-continuous arterial spin labeling (pCASL), a perfusion-weighted MRI technique, was used. MRI scans were conducted at baseline within 4 weeks of randomization as well as following the 12-month intervention. All MRI data were acquired with a Siemens 3.0 Tesla Verio Scanner (Magnetom Verio, Munich, Germany). A 32-channel head coil was used for radio frequency (RF) transmission and reception. Foam padding was positioned within the head coil to minimize patient motion. A high-resolution T1-weighted anatomical image was acquired for co-registration with the following sequence parameters: Magnetization Prepared Rapid Acquisition of Gradient Echo (MPRAGE), matrix = 256, field-of-view (FOV) = 250 mm, voxel size = 1.0 × 1.0 × 1.0 mm, slices = 192 (sagittal plane, acquired left to right), slice thickness = 1.0 mm, repetition time (TR) = 1,900 ms, echo time (TE) = 2.93 ms, inversion time (TI) = 900 ms, flip angle = 9°, sequence duration = 4:26 min. Perfusion-weighted images were collected using a multi-slice pCASL protocol for perfusion quantification with the following parameters (Jung et al., 2010): matrix size = 64, FOV = 220 mm, voxel size = 3.40 × 3.40 × 5.0 mm, slices = 20 (axial plane, acquired in ascending order), slice thickness = 5.0 mm, gap between slices = 1 mm, single slice acquisition time = 48 ms, label duration = 1,500 ms, post-label delay = 1,500 ms, TR/TE = 4,090/21 ms, volumes = 80, number of label/control pairs = 40, flip angle = 90°, RF blocks = 80, RF pulses = 20, gap between pulses = 360 μs, bandwidth = 2,298 Hz/Px, and sequence duration = 5:35 min.

### Statistical analyses

#### Preprocessing and perfusion quantification

The preprocessing and perfusion quantification was performed as described in Stillman et al. (2021). Briefly, once reconstructed, the time series was realigned using the middle volume as a reference and co-registered with the T1-weighted anatomy (flirt; FMRIB Software Library version 5.0.9, Oxford, United Kingdom). The time series was subsequently visually inspected for proper alignment. Partial volume estimates were derived from the T1-weighted anatomy using FSL’s Fully Automated Segmentation Toolbox. Such high-resolution tissue-type maps were used for partial volume correction, nuisance signal regression, and tissue-specific perfusion quantification. FSL’s Bayesian Inference for Arterial Spin Labeling toolbox (BASIL) was used for quantification of perfusion. A rCBF image was generated from the co-registered time-series using pairwise tag-control subtraction (asl_file). The images were adjusted for slice-time delay. Then, BASIL’s oxford_asl command was run with the partial volume and spatial correction options turned on to further control for spurious signals. The cerebral spinal fluid image from FAST was designated as the tissue reference for nuisance regression. This step also corrected the proton density image to adjust for potential errors in the blood brain partition coefficient and applied a ventricular mask to the corrected image to isolate and compute the magnetization equilibrium (M0) of the white and gray matter tissue. The M0 value was used to approximate the M0 of the arterial blood and convert the relative rCBF values into absolute units of ml/100 g/min (asl_calib). Finally, the calibrated images were normalized to MNI space. Volumes from each person, from each time point, were combined into 4D files for group level statistical analyses. This resulted in a baseline and follow-up rCBF images for each participant.

#### Comparison of demographic variables based on fat mass and obesity associated gene genotype

Before conducting analyses addressing the primary and secondary goals of this study, independent samples *t-*tests and chi square analyses were conducted to examine differences in BMI and other potentially confounding variables between FTO A carriers and T/T homozygotes (Table 1). Given the low minor allele (A) frequency, previous studies on FTO have compared A carriers to T/T homozygotes, which is also the approach being undertaken in the present study. We also examined differences between the full WIN sample (*N* = 125) and the 105 participants who had both useable genetic and perfusion data using *t-*tests or chi squares (Table 2). The sample size of AT/AA and the TT allele groups with both usable MRI and genetic data were 70 and 35, respectively. A Hardy-Weinberg equilibrium test was then conducted to determine expected allelic distribution in the sample.

**TABLE 1:** Baseline comparisons of FTO T Homozygotes and A Carriers.

Baseline measure	All participants (*M* ± *SD*) *N* = 105	A carriers (*M* ± *SD*) *N* = 70	T/T homozygotes (*M* ± *SD*) *N* = 35	*p* *
Age (years)	44.90 (± 8.18)	45.83 (± 7.30)	43.06 (± 9.55)	0.10
Sex	26 male	17 male	9 male	0.87
Race	24 non-Caucasian	19 non-Caucasian	5 non-Caucasian	0.11
BMI (kg/m^2^)	32.42 (± 3.92)	32.82 (± 3.99)	31.59 (± 3.69)	0.09
Systolic blood pressure (mmHg)	118.70 (± 11.81)	119.83 (± 10.42)	116.45 (± 14.07)	0.17
Diastolic blood pressure (mmHg)	71.55 (± 9.11)	71.93 (± 8.62)	70.79 (± 10.11)	0.55
Waist circumference (cm)	106.37 (± 10.08)	106.86 (± 9.44)	105.38 (± 11.32)	0.48
Resting heart rate (beats per minute)	66.03 (± 9.04)	65.63 (± 9.02)	66.82 (± 9.17)	0.53

*Demographic differences between TT homozygotes and A carriers were assessed using t-tests (continuous variables) or chi square (categorical variables). The *p*-values represent the comparisons between the A carriers and the TT homozygotes.

**TABLE 2:** Baseline demographic comparison of full WIN sample to present subsample with available genetic and pCASL data.

	Present sample (*M* ± *SD*) *N* = 105	Full WIN sample (*M* ± *SD*) *N* = 125	p*
Age (years)	44.90 (± 8.17)	41.65 (± 10.28)	0.063
Sex	26 male	27 male	0.049
Education (years)	16.49 (± 2.65)	15.70 (± 2.67)	0.739
Systolic BP (mmHg)	118.70 (± 11.81)	117.97 (± 11.17)	0.671
Diastolic BP (mmHg)	71.55 (± 9.11)	69.10 (± 7.40)	0.191
Waist circumference (cm)	106.37 (± 10.12)	105.25 (± 7.56)	0.101
Resting heart rate (bpm)	66.25 (± 8.98)	68.05 (± 12.47)	0.069
Weight (kg)	92.01 (± 14.51)	88.05 (± 11.15)	0.115
Baseline BMI (kg/m^2^)	32.39 (± 3.96)	32.40 (± 3.96)	0.925

*Demographic differences between samples were assessed using t-tests (continuous variables) or chi square (categorical variables).

#### Comparison of baseline resting cerebral blood flow based on fat mass and obesity associated gene genotype

To test for differences in rCBF at baseline between FTO A carriers (AT/AA) and non-carriers (T/T homozygotes), a general linear model in FSL (glm_gui) was created. Age, sex and BMI were included as covariates and genotype (A carrier, TT homozygote) was included as an explanatory variable of interest. Age and sex were chosen as covariates *a priori* because each are known to affect cerebral hemodynamics along with other brain health outcomes (Matteis et al., 1998; Ghisleni et al., 2015; Lotze et al., 2019). In addition, since we expected that BMI might partly covary with FTO alleles, we included BMI as a covariate to attempt to isolate the effect of FTO allele variants independently from BMI. *F*-tests were conducted to identify brain regions where rCBF differed between A carriers and T/T homozygotes.

#### Comparison of intervention-related changes in resting cerebral blood flow based on fat mass and obesity associated gene genotype

To examine whether FTO moderated intervention-related changes in rCBF, the rCBF change maps (Stillman et al., 2021) were entered into a general linear model in FSL BASIL, controlling for age, sex, and BMI. FTO genotype and intervention group were entered as the explanatory variables of interest. Since by chance there were few people in the Diet + MODEX group, the Diet + MODEX and diet + HIGHEX groups were combined into an “exercise exposure” group as in Stillman et al. (2021). *F*-tests were conducted to identify clusters where rCBF changes differed between FTO groups (i.e., A carrier > TT and TT > A carrier contrasts) or as a function of both FTO and intervention groups (i.e., a FTO × intervention group interaction). This is statistically equivalent to testing for a time × FTO, and a time × FTO × intervention group interaction, respectively.

All results were corrected for multiple comparisons using the FSL cluster command, which estimated using Gaussian Random Field (GRF) theory that contiguous clusters ≥ 293 voxels and *z*-values > 2.3 survived a threshold of *p* < 0.05. We extracted mean perfusion values from significant clusters in order to visualize the results and the direction of any significant interactions. We also compared rCBF in A carriers relative to TT homozygotes by dividing mean perfusion from significant clusters in A carriers by that of TT carriers.

## Results

### Demographic characteristics of participants

Analysis of baseline demographic data was performed using independent *t*-tests and Chi-square analyses. No significant difference between FTO groups existed for age and sex (Table 1). However, there was a marginal difference (*p* = 0.09) in BMI between T homozygotes and A carriers, such that T homozygotes had numerically lower BMI than A carriers (Table 1). When comparing the full WIN sample with the subsample with both genetic and perfusion data included in our study, we found that there was a smaller percentage of females in our sample (75%) compared to the full sample (78% female). No other demographic characteristics were significantly different between the full and present sample.

### Allelic frequency of sample

A Hardy-Weinberg equilibrium test showed that the sample had a minor allele frequency of 56% (X^2^ = 0.90, *p* = 0.34; A/A: *n* = 23, A/T: *n* = 47, T/T: *n* = 35) which is not significantly different from that expected by Hardy-Weinberg equilibrium.

### Fat mass and obesity associated gene explains variance in baseline resting cerebral blood flow

**FIGURE 1:**
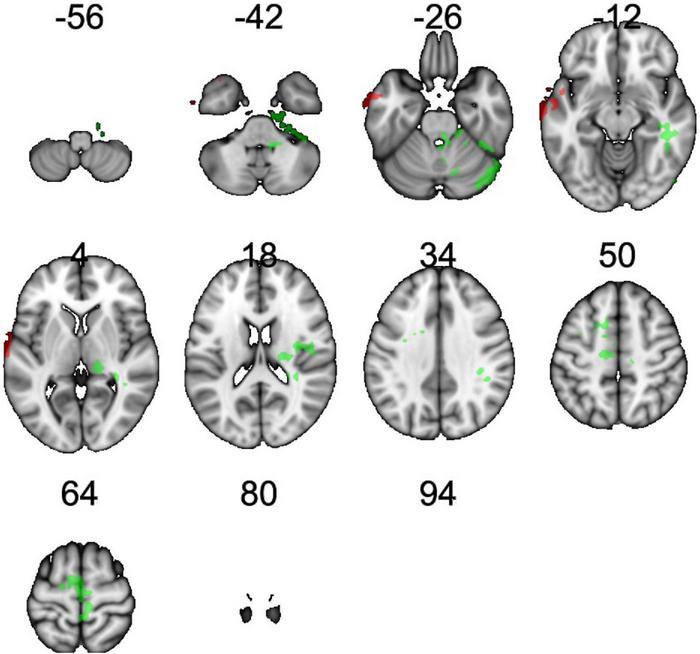
Regions showing an effect of FTO on resting cerebral blood flow (rCBF). Green indicates regions where A carriers had greater rCBF than TT homozygotes (A carrier > T/T contrast), and red indicates regions showing greater perfusion in TT homozygotes than A carriers (T/T > A carrier contrast). Results were corrected for multiple comparisons. It was determined that contiguous clusters ≥ 293 voxels and *z*-values > 2.3 survived a threshold of *p* < 0.05.

**FIGURE 2:**
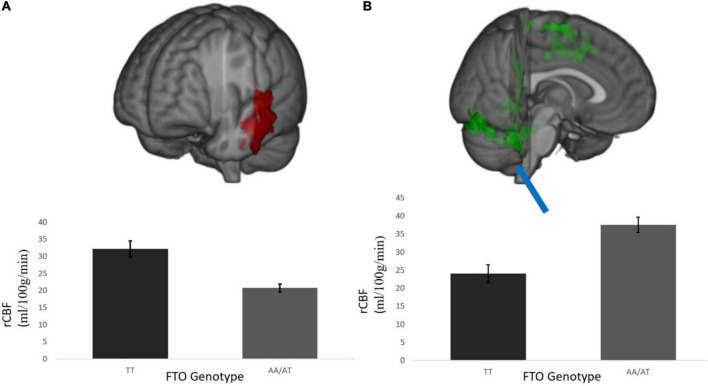
**(A)** Temporal cluster where rCBF for A carriers < T/T homozygotes and **(B)** representative cluster in the posterior occipital cortex where rCBF for A carriers > T/T homozygotes; This was a diffuse cluster had peak coordinates in the occipital lobe, extending into cerebellum, brain stem, and motor cortex. rCBF values are measured in ml/100 g/min. Results were corrected for multiple comparisons and was estimated that contiguous clusters ≥ 293 voxels and *z*-values > 2.3 survived a threshold of *p* < 0.05.

rCBF scans at baseline and follow-up were acquired successfully on 109 participants. Of these, 107 were of usable quality following preprocessing. Of the 107 participants, 105 had usable FTO genetic data. The final sample size for the present analyses is therefore *N* = 105 (below 2% failure rate). Five clusters showed a significant difference between A carriers and T/T homozygotes at baseline (Figure 1 and Table 3). These were large clusters spanning temporal, occipital, motor, and brain stem regions. T/T homozygotes had greater rCBF in a cluster located in the temporal pole extending into the anterior middle temporal gyrus, while A carriers had greater rCBF in a cluster located in the lateral occipital cortex extending into motor cortex and brain stem (Figures 2A,B). These differences reflect a 32% decrease in mean rCBF in FTO A carriers in the left temporal lobe, and a 52% increase in mean rCBF in the right occipital, motor, and brainstem clusters compared to T/T homozygotes.

**TABLE 3:** Clusters showing main effect of the FTO group (T/T homozygote vs. A carrier) on resting cerebral perfusion (rCBF).

Contrast	Cluster ID	Peak MNI (x, y, z)	Cluster size (k)	Peak Z (Max)	Primary brain regions	Mean CBF (ml/100 g/min)
***TT* > *AA/AT***	*1*	79, 59, 30	777	3.65	Middle temporal gyrus anterior division	32.1738 TT 20.752 AT/AA
***AA/AT* > *TT***	*2*	26, 48, 50	984	3.47	Right cerebral white matter	34.805 TT 43.494 AT/AA
***AA/AT* > *TT***	*3*	51, 55, 59	950	3.9	Left cerebral white matter; left cerebral cortex Precentral gyrus; supplementary motor cortex	37.387 TT 49.1648 AT/AA
***AA/AT* > *TT***	*4*	35, 47, 21	846	3.38	Occipital extending into brain stem	25.866 TT 32.5812 AT/AA
						

### Fat mass and obesity associated gene and treatment group assignment do not moderate intervention-related changes in resting cerebral blood flow

Of the *n* = 35 T/T homozygotes in the sample, *n* = 17 were in the Diet only group and *n* = 18 were in the exercise exposure group. Of the *n* = 70 A carriers, 26 were in the Diet only group and *n* = 44 were in the exercise exposure group. The proportion of A carriers between the diet only and DIET + Exercise group was not significantly different, X^2^ (2, 105), = 1.20, *p* = 0.36. The 12-month weight loss intervention increased mean rCBF in distributed regions throughout the cortex. However, we found no evidence that intervention-related changes in rCBF were modified by FTO genotype nor by the combined effect of intervention group and FTO genotype (i.e., no significant time × FTO, or time × FTO × group interactions).

## Discussion

The distribution frequency of the FTO allele in our sample was similar to that reported in previous literature (Ho et al., 2010b), but slightly higher than that reported for populations of European descent. This slight difference could be related to the mixed race/ethnic demographics in this sample or the targeted recruitment of individuals with overweight or obesity. Carriers of the obesity risk-conferring A allele had a slightly higher BMI at baseline than T/T homozygotes (albeit not significantly), consistent with previous literature. Supporting our initial hypothesis, rCBF varied as a function of the FTO allele across several brain regions in adults with overweight or obesity. We observed a decrease in rCBF in A carriers in temporal regions, and an increase in rCBF in occipital, motor, and brainstem regions, compared to T/T homozygotes. Although differences in rCBF were not detected in the frontal lobe as predicted, A carriers showed a reduction in rCBF in the left temporal lobe relative to T/T homozygotes. The *greater* rCBF in clusters located in the occipital lobe, motor cortex and brainstem for A carriers was surprising since decreases in other aspects of brain integrity, such as gray matter volume, have been found in A carriers (e.g., Ho et al., 2010b). Further, intervention-induced changes in rCBF were not moderated by allelic variation in FTO. Overall, these results indicate that (a) individual variation in rCBF within a sample with overweight and obesity can be attributed to a common FTO variant, but (b) a weight loss intervention is equally effective at increasing rCBF, regardless of FTO genotype.

Our results suggest a complex relationship between the FTO variant and rCBF in individuals with overweight and obesity, such that rCBF appears to be higher in T/T homozygotes compared to A carriers in some brain regions, but lower in others. The regions showing differences in rCBF between FTO A carriers and T homozygotes overlap with literature showing decreases in gray matter volume in both the temporal and occipital lobes as a function of BMI and FTO (Taki et al., 2008; Ho et al., 2010b). One question that arises from this regional overlap is whether lower volumes in these regions with higher BMI are at least partially explained by this FTO variant (Willeumier et al., 2011). The convergence of these regions may also be physiologically significant as they are implicated in the etiology of obesity, diabetes, and Alzheimer’s disease (Daulatzai, 2017). However, our finding of higher rCBF in FTO A carriers in several regions (occipital, motor cortex, brainstem) was surprising since there is evidence for cortical atrophy in these same regions for A carriers (Ho et al., 2010b). There could be several reasons to explain this pattern including significant differences in the age and weight status of Ho et al. (2010b) sample compared to the demographics of this sample.

The mechanisms underlying increased perfusion among A carriers is a matter of speculation. It is possible that the molecular pathways initiated by the genetic variant may be acting differently across the brain (occipital vs. temporal). One possible molecular mechanism relates to the known role of FTO in homeostatic processes related to energy regulation and body weight, such as mitochondrial thermogenesis, adipogenesis, and caloric intake (Ganeff et al., 2019). Prior investigations of FTO show that with *in situ* hybridization in mice, FTO is highly expressed in feeding-related cell nuclei in the brain, specifically in regions such as the brainstem and hypothalamus (Fredriksson et al., 2008). If such differences in expression are also found in occipital and temporal lobe areas, it might explain the differential patterns in rCBF at baseline that we report here. The expression of FTO in neurons and correlations with elevated BMI and altered cerebral perfusion could also highlight cerebral pathways and biological mechanisms that influence rCBF, such as control of energy homeostasis or macronutrient intake (Morton et al., 2006). Nonetheless, these points remain points of speculation as we have a poor understanding of whether the molecular products of energy regulation influence rCBF patterns or whether FTO is differentially expressed in the regions we identify here.

Importantly, we found that neither variation in the FTO polymorphism, nor the combined influence of FTO and intervention group, moderated 12-month changes in rCBF. This suggests that the rCBF response to the intervention was not moderated by FTO genotype and expands upon our previous work in the same (albeit slightly larger) sample in which genetic data were not considered (Stillman et al., 2021). Therefore, regardless of genotype, individuals with overweight and obesity demonstrate comparable increases in cerebral perfusion resulting from weight loss. Further investigation into the mechanisms and genetic moderators contributing to obesity and rCBF may be beneficial in determining the efficacy of lifestyle interventions to improve brain health across the lifespan.

It is important to speculate about the behavioral significance of these findings and whether FTO-related differences in rCBF in these regions translates to either increased risk for emotional, cognitive, or neurologic conditions. Since our study was in a relatively young sample we do not know whether these differences in rCBF will likely translate to more accelerated cognitive losses with increasing age, but most evidence suggests that FTO is not a common polymorphism associated with accelerated cognitive decline with only a single study linking the polymorphism to verbal fluency in elderly men with overweight and obesity (Benedict et al., 2011). It will be critical for future research to link FTO-related differences in rCBF with behavioral or disease measures in order to enhance interpretation and meaning to these patterns.

The results of our study should be interpreted considering some limitations. First, while our sample size of 125 participants with overweight and obesity is reasonably large for a neuroimaging study, it is quite small for a genetic study. Differences in neuroimaging metrics, including rCBF, may be endophenotypes more proximal to molecular products of genetic variation than other outcomes (e.g., behavior) (Nikolova and Hariri, 2015; Thompson et al., 2017), reducing the sample size necessary to detect effects. Nonetheless, larger studies are necessary to further elucidate the association between the FTO SNP and rCBF, as well as the potential moderating effects of FTO in intervention-induced changes in rCBF. Our intervention groups did not have equal sample sizes. This was due to the nature of the neuroimaging ancillary and that participants were volunteers who were enrolling into the parent intervention on weight loss. Thus, a randomized clinical trial in which participants are allocated equally and randomly across groups would help interpret the outcomes from the intervention data. Along these lines, clearer patterns may also emerge by expanding the range of BMI. The participants in this study all had overweight or obesity. It is therefore unclear as to whether our results would generalize to a healthy weight sample. Finally, we examined only one FTO polymorphism (rs9939609) in this study. FTO rs9939609 is an established obesity risk factor and is the most widely studied SNP of this gene, but other FTO polymorphisms may also contribute to BMI or rCBF independently or in combination with FTO rs9939609. Larger samples and expanded genetic testing may allow for an examination of multiple polymorphisms (e.g., haplotypes) in order to better understand the association between FTO and the brain.

## Conclusion

Despite these limitations and unexpected findings, this is one of the largest studies so far examining associations between FTO and BMI using a sensitive measure of rCBF. As such, there are some broad conclusions that can be drawn from these results. First, there is an association between the FTO SNP rs9939609 and cerebral perfusion across a diffuse set of brain regions, independent of age, sex, and BMI. We also found that despite baseline differences in rCBF as a function of the FTO polymorphism, FTO did not modify weight loss-induced changes to rCBF. This suggests that, regardless of genotype, individuals with overweight and obesity demonstrate comparable improvements in cerebral perfusion in response to a weight loss intervention.

## Data availability statement

The original contributions presented in the study are publicly available. This data can be found here: https://doi.org/10.6084/m9.figshare.20514162.v1.

## Ethics statement

The studies involving human participants were reviewed and approved by the University of Pittsburgh Institutional Review Board. The patients/participants provided their written informed consent to participate in this study.

## Author contributions

JJ, KE, KR, and RR contributed to conception and design of the study. CS and KR organized the database. CS, CK, and GB performed the statistical analysis. GB wrote the first draft of the manuscript. CS and CK wrote sections of the manuscript. All authors contributed to manuscript revision, read, and approved the submitted version.
